# CEBPα/miR-101b-3p promotes meningoencephalitis in mice infected with *Angiostrongylus cantonensis* by promoting microglial pyroptosis

**DOI:** 10.1186/s12964-023-01038-y

**Published:** 2023-02-06

**Authors:** Xingda Zeng, Jia Shen, Dinghao Li, Shurui Liu, Ying Feng, Dongjuan Yuan, Lifu Wang, Zhongdao Wu

**Affiliations:** 1 Department of Parasitology of Zhongshan School of Medicine, Sun Yat-Sen University, Guangzhou, 510080 China; 2 Guangzhou Key Laboratory for Clinical Rapid Diagnosis and Early Warning of Infectious Diseases, KingMed School of Laboratory Medicine, Guangzhou Medical University, Guangzhou, 510180 China; 3 Key Laboratory of Tropical Disease Control, Ministry of Education, Sun Yat-Sen University, Guangzhou, 510080 China; 4 Provincial Engineering Technology Research Center for Biological Vector Control, Guangzhou, 510080 China; 5 School of Medicine, South China University of Technology, Guangzhou, 510006 China; 6 College of Veterinary Medicine, South China Agricultural University, Guangzhou, 510642 China

**Keywords:** *Angiostrongylus cantonensis*, CEBPα, miR-101b-3p, Meningoencephalitis, Microglial pyroptosis

## Abstract

**Background:**

*Angiostrongylus cantonensis* (*A. cantonensis*) infection can induce acute inflammation, which causes meningoencephalitis and tissue mechanical injury to the brain. Parasite infection–induced microRNAs play important roles in anti-parasite immunity in non-permissive hosts. miR-101b-3p is highly expressed after *A. cantonensis* infection; however, the role of miR-101b-3p and the transcription regulation of miR-101b-3p in *A. cantonensis* infection remain poorly characterized.

**Results:**

In the present study, we found that miR-101b-3p inhibition alleviated inflammation infiltration and pyroptosis in *A. cantonensis* infection. In addition, we found that CCAAT/enhancer-binding protein alpha (CEBPα) directly bound to the − 6-k to − 3.5-k region upstream of miR-101b, and CEBPα activated miR-101b-3p expression in microglia. These data suggest the existence of a novel CEBPα/miR-101b-3p/pyroptosis pathway in *A. cantonensis* infection. Further investigation verified that CEBPα promotes pyroptosis by activating miR-101b-3p expression in microglia, and microglial pyroptosis further promoted inflammation.

**Conclusions:**

Our results suggest that a CEBPα/miR-101b-3p/pyroptosis pathway may contribute to *A. cantonensis* infection–induced inflammation and highlight the pro-inflammatory effect of miR-101b-3p.

**Video Abstract**

**Supplementary Information:**

The online version contains supplementary material available at 10.1186/s12964-023-01038-y.

## Background

*Angiostrongylus cantonensis*, first found in 1933 by Chen Xintao in Guangzhou, China, is a common foodborne parasite [1]. A large number of cases have been documented in tropical and subtropical countries and regions, which are the natural epidemic foci for this infection, but, with the process of globalization, worldwide tourism and business exchanges have led to the appearance of Angiostrongyliasis in Europe, which is not a natural epidemic focus [2]. Rats are the permissive host of *A. cantonensis* in which larvae migrate from the brain to the lungs and then develop into adult worms [3]. On the contrary, human and mice are non-permissive hosts of *A. cantonensis*. After infection with *A. cantonensis*, humans show severe central nervous symptoms, including nerve demyelination, neck stiffness, and severe head pain [1].

Mebendazole and albendazole are commonly used as therapeutics for *A. cantonensis* infection[4] but may lead to the aggravation of central nervous system symptoms[5, 6], and albendazole can also lead to neutropenia due to myelosuppression[7]. Side effects from praziquantel, the classic anti-parasitic drug, were also reported to include headache, nausea, abdominal pain, diarrhea, limb fatigue, palpitations, and chest tightness[8, 9]. Long-term usage of glucocorticoids may lead to immunocompromise, diabetes, hypertension, and glaucoma[10, 11]. Therefore, there is an urgent need to find new treatments. Elucidation of the pathogenesis of *A. cantonensis* infection will provide a basis for finding new drug targets.

Our previous study found that miR-101b-3p is significantly up-regulated in the brains of *A. cantonensis*–infected mice and destroys the antioxidant system of *A. cantonensis* by targeting *A. cantonensis’ SOD3* gene to achieve a parasite-elimination effect [12]. The function and activation of miR-101b-3p, which mediates the host–parasite interaction, are worth further exploration. In previous research, miR-101 was reported to be a pro-inflammatory factor, and miR-101-3p targets TRIB1, leading to pro-inflammatory chemokine CXCL8 secretion [13]. Further, miR-101 promotes hypersensitivity and an inflammatory response and aggravates neuropathic pain by targeting MKP-1 in rat microglia [14], while the inhibition of miR-101 by Mirt2/PI3K/AKT alleviated cardiac structure and function impairments [15]. At this time, however, the function of miR-101 in *A. cantonensis* infection is unclear.

In the present study, we found different expressions of miR-101-2-3p in rats infected with *A. cantonensis* and miR-101b-3p in mice infected with *A. cantonensis*, respectively. We then investigated the pro-inflammatory role of miR-101b-3p in *A. cantonensis* infection and the contribution to meningoencephalitis. We found that pyroptosis in the mouse brain was increased after *A. cantonensis* infection, and miR-101b-3p blocking lead to a decrease in inflammasomes. We further demonstrated that microglia expressing miR-101b-3p in *A. cantonensis–*infected mice are activated by CCAAT/enhancer-binding protein alpha (CEBPα). Meanwhile, miR-101b-3p inhibition reduced CEBPα-induced microglia pyroptopsis. The findings of the present study suggest that miR-10b-3p could be used as a potential target for the treatment of meningoencephalitis.

## Methods

### Animals and ethics

Male BALB/c mice were purchased from Guangdong Medical Laboratory Animal Centre. To induce infection, mice were intake Angiostrongylus cantonensis by gavage needle administration. All animal experiments were approved by the Sun Yat-sen University Committee for Animal Research and conformed to the Guidelines for the Care and Use of Laboratory Animals of the National Institute of Health in China.

### *A. cantonensis* infection model

Third-stage larvae of *A.cantonensis* were collected from infected experimental *Biomphalaria glabrata* snails. Snail tissue were isolated and incubated in 1% HCl and 1% pepsin solution at 37 °C for 40 min. 6–8 week age mice and rat were used and intragastric administration with *A.cantonensis* third-stage larvae. Infection dose of 30 larvae/mouse and 200 larvae/rat were used as *A.cantonensis* infection model in this research.

### Real-time polymerase chain reaction (RT-PCR) analysis

Total RNA was isolated from fresh brain tissue and BV2 cells using TRIzol reagent (Invitrogen, Carlsbad, CA, USA) according to manufacturer’s instructions. The Mir-X miRNA First-strand Synthesis Kit (Takara Bio, Shiga, Japan) was used for microRNA (miRNA) reverse transcription. The Evo M-MLV RT Premix and SYBR^®^ Green Premix Pro Taq HS qPCR Kit (Accurate Biotechnology Co., Ltd., Hunan, China) were used for quantitative PCR (qPCR) determination of messenger RNA (mRNA) and miRNA expression levels, GAPDH and U6 snRNA were used as internal controls, and the 2^−ΔΔCT^ method was used for fold-change calculation. The primers used in the assay are shown in Table 1.

**Table 1:** Primers of qPCR

Target name	Forward primer	Reverse primer
Mmu-miR-101b-3p Rno-miR-101–2-3p	GGGCTACTGTGATAGCTAAAA GGGCTACTGTGATAGCTAAAA	mRQ 3′Primer in Takara kit mRQ 3′Primer in Takara kit
CEBPα	CAAGAACAGCAACGAGTACCG	GTCACTGGTCAACTCCAGCAC
NLRP3	ATTACCCGCCCGAGAAAGG	TCGCAGCAAAGATCCACACAG
CEBPβ	CTTCCTCTCCGACCTCTTCG	AGGCTCACGTAACCGTAGTC
IL-18	GACTCTTGCGTCAACTTCAAGG	CAGGCTGTCTTTTGTCAACGA
IL-1β	GCAACTGTTCCTGAACTCAACT	ATCTTTTGGGGTCCGTCAACT
ASC	CTTGTCAGGGGATGAACTCAAAA	GCCATACGACTCCAGATAGTAGC
GSDMD	CCATCGGCCTTTGAGAAAGTG	ACACATGAATAACGGGGTTTCC
Caspase1	ACAAGGCACGGGACCTATG	TCCCAGTCAGTCCTGGAAATG
TNFα	CCCTCACACTCAGATCATCTTCT	GCTACGACGTGGGCTACAG
IFNγ	ATGAACGCTACACACTGCATC	CCATCCTTTTGCCAGTTCCTC
IL-6	CTGATGCTGGTGACAACCAC	CAGAATTGCCATTGCACAAC
RCL1	GCGCACTCACTCAGCTACG	GGCTGGTAGTATAAGGTTGTTCC

### Western blot analysis

Brain tissue and BV2 cells were homogenized with radioimmunoprecipitation assay lysis buffer (Thermo Fisher Scientific, Waltham, MA, USA) containing protease and phosphatase (Thermo Fisher Scientific) individually; then, lysate suspensions were incubated on ice for 10 min and centrifugated at 12,000 g. The supernatant was next subjected to 12% sodium dodecyl sulfate–polyacrylamide gel electrophoresis at 80 V for 3 h and transferred onto polyvinylidene difluoride (PVDF) membranes (Millipore, Burlington, MA, USA). Five percent non-fat milk was used for antigen blocking of PVDF membranes, which were incubated with primary antibody overnight, then with secondary antibody before band image capture. Band images on the PVDF membranes were captured and analyzed using the ChemiDoc Touch System (BioRad Laboratories, Hercules, CA, USA). The primary antibodies against CEBPα (Abcam, Cambridge, UK), CEBPβ (Abcam), IL-18 (Abcam), IL-1β (Cell Signaling Technology, Danvers, MA, USA), GSDMD (Abcam), NLRP3 (Cell Signaling Technology), caspase-1 (Cell Signaling Technology), and GAPDH (Cell Signaling Technology) and horseradish peroxidase–conjugated secondary antibody (Abcam) were used in this study.

### Cell culture and gene transfection

Mouse microglia BV2 cells were cultured in high-glucose Dulbecco’s modified Eagle’s medium supplemented with 10% fetal bovine serum (Thermo Fisher Scientific). Medium was combined with 100 U/mL of penicillin and 100 mg/mL of streptomycin. The cells mentioned above were kept at 37 °C with 5% CO_2_ in a humidified incubator.

Stable and transient transfections were performed. miRNA antagomir (RiboBio, Guangzhou, China) and GV146-CEBPΑ vector (GeneChem, Daejeon, South Korea) were used and transfected into BV2 cells using the Lipo3000 Kit (Invitrogen). Transfection was observed by fluorescence microscopy. BV2 cells were subsequently cultured for 12 and 48 h for qPCR and western blot detection.

### Adeno-associated virus (AAV) administration

Caudal venous administration of AAV2/PHP.eB (1.5 × 1011 v.g./mouse) was performed in 6-week-old BalB/C mice. The green fluorescent protein (GFP) expression of AAV in mice was observed using fluorescence microscopy.

### Dual-luciferase reporter assay

We transfected 293 T cells with the pGL3-basic, pGL3-pro1, pGL3-pro2, and PT-m-CEBPα plasmids using RNAiMAX (Invitrogen). Then, 48 h after transfection, Luciferase Assay Reagent II (Promega Corporation, Madison, WI, USA) was added to the plates after cell lysis and we detected Firefly luciferase, then added Stop & Glo^®^ Reagent (Promega Corporation) and detected Renilla luciferase. Luciferase activities were detected using the Infinite F500 Multimarker Analyzer (Tecan, Männedorf, Switzerland).

### Immunofluorescence analysis

Brian tissues were fixed in 4% paraformaldehyde and embedded in paraffin. Tissue slides were dewaxed and incubated with primary antibodies against CEBPα (Cell Signaling Technology), cleaved IL-1β (Cell Signaling Technology), and NLRP3 (Cell Signaling Technology) overnight at 4 °C. The slides were subsequently incubated with the appropriate secondary antibodies at room temperature, then stained with Iba1 (synaptic systems, Germany) for 1 h and 4′,6-diamidino-2-phenylindole (DAPI) (Elabscience Biotechnology Co., Ltd.) for 5 min at room temperature. We used 1% Triton X-100 solution (Sigma,USA) as a cell membrane permeabilizer.

### Neurological severity scores (NSS)

1. 0 score: normal walk—no neurological impairment.
2. 1 score: one forelimb flexion during tail lifting—slight neurological impairment.
3. 2 score: inability to walk straight—mild or moderate neurological impairment.
4. 3 score: obvious incline to one side or circling when walking—moderate neurological impairment.
5. 4 score: inability to walk spontaneously, diminished consciousness—severe neurological impairment.
6. 5 score: death due to infectious inflammation

### Statistical analysis

All data were statistically analyzed using SPSS 16.0, and the measurement results were expressed as mean ± standard error (mean ± SD). One way ANOVA and student’s t test were used to analyze the differences between the groups. Pearson correlation analysis was used to analyze correlation between two gene expression. *P* < 0.05 was statistically significant.

## Results

### Mmu-miR-101b-3p was a pro-inflammatory factor in meningoencephalitis caused by A. cantonensis infection

**Fig. 1:**
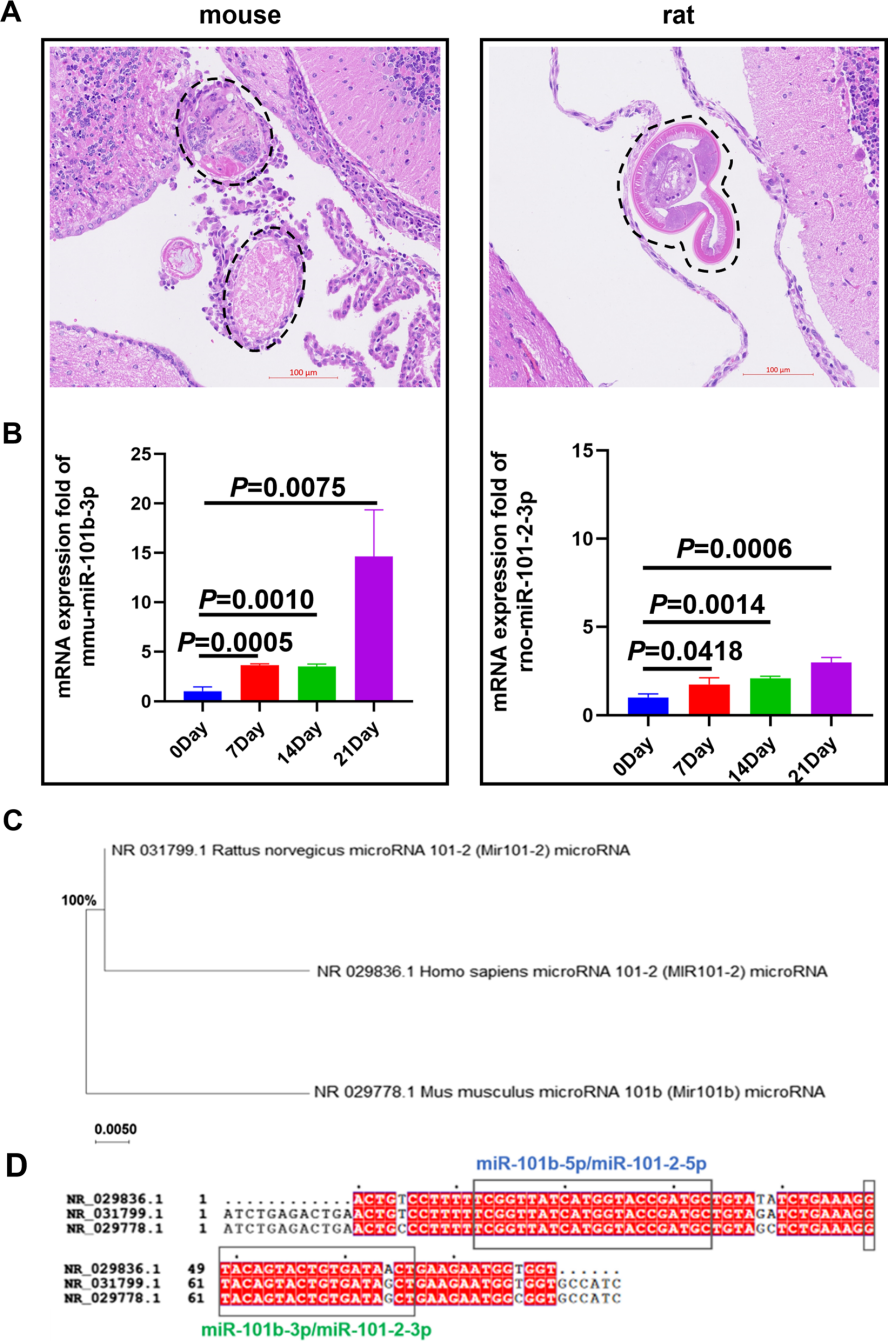
miR-101b-3p is expressed in permissive and non-permissive hosts. **A** Larvae in the brains of mice and rats. **B** miR-101b-3p was more highly expressed in mouse brains than rat brains. **C, D** Evolutionary tree and sequence analyses revealed that miR-101b/miR-101-2 in mice, rats, and humans is highly homologous

Previously, our research revealed that miR-101b-3p interrupted the *A. cantonensis* antioxidant system by targeting the *SOD3* gene of *A. cantonensis* and therefore eliminating the worm [12]. At the same time, highly expressed miR-101b-3p may led to antigen shedding of *A. cantonensis*, which may stimulate inflammatory infiltration. *A. cantonensis* achieves different parasitism outcomes in rats (permissive hosts) and mice (non-permissive hosts); the worms develop sturdily in rats but are degraded in mice and surrounded by inflammatory infiltration (Fig. 1A). We examined miR-101b-3p expression levels in rats and mice and found that miR-101b-3p was highly expressed in both animals’ brains after *A. cantonensis* infection, but the miR-101b-3p up-regulation trend in mice was more intense than that in rats (Fig. 1B). MiR-101b in mice, which is also known as miR-101–2 in rats and humans, is highly homologous (Fig. 1C and D), suggesting that miR-101b/miR-101–2 has same function in *A. cantonensis* infection, and miR-101b-3p might be an important factor in meningoencephalitis caused by A. cantonensis infection.

**Fig. 2:**
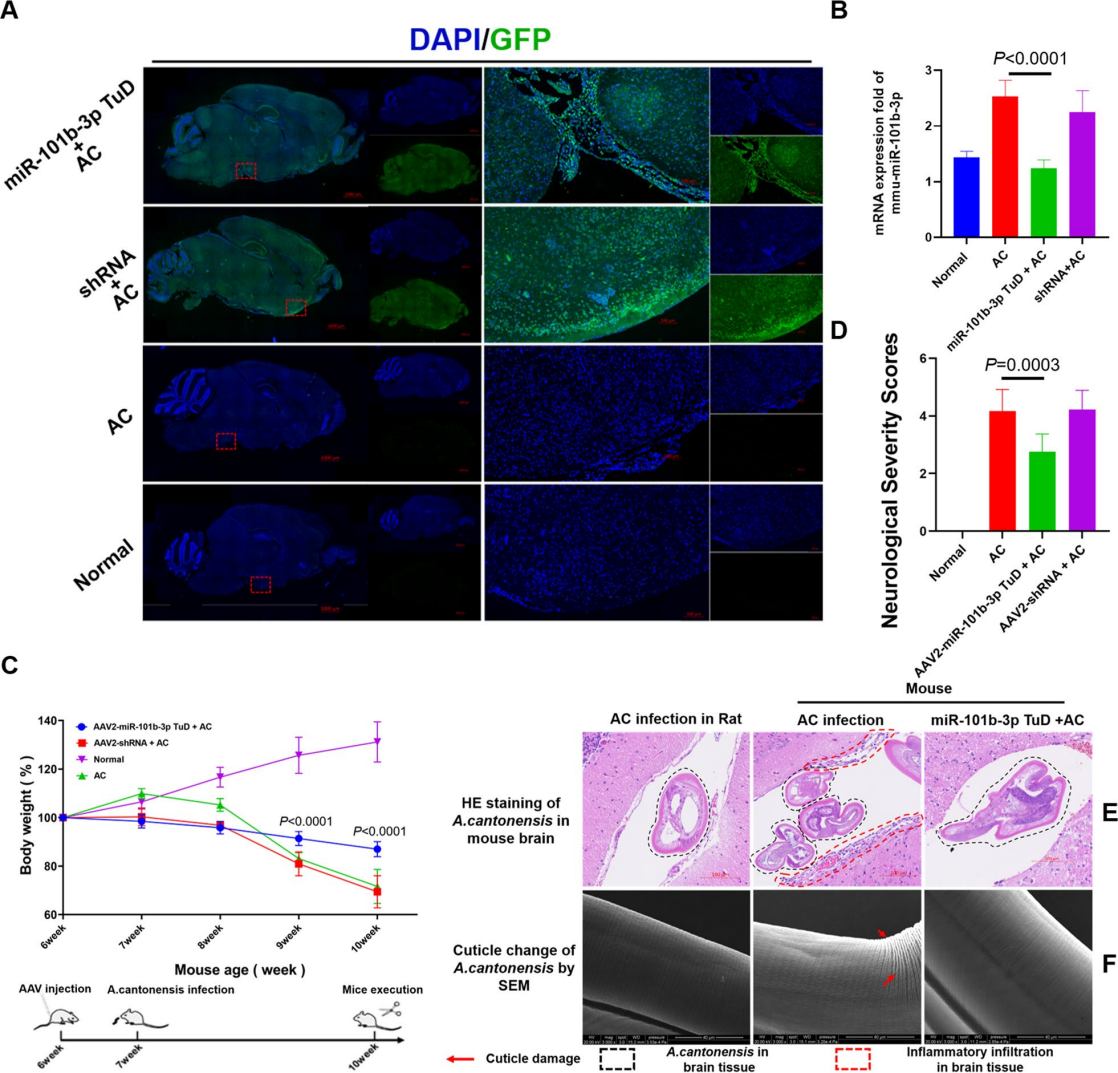
miR-101b-3p is an important pro-inflammatory factor in *A. cantonensis* infection. **A** AAV infection efficiency of the mouse brain; GFP indicates the area infected by the AAV. **B** Brain miR-101b-3p level in mice; the miR-101b-3p level was decreased after AAV administration. **C** The body weight of mice was alleviated after AAV administration.** D** Neurological severity scores were reduced after miR-101b-3p inhibition. **E** Inflammatory infiltration around larvae was alleviated after miR-101b-3p inhibition. **F** Cuticle damage of stage IV larvae in mouse brains was alleviated after miR-101b-3p inhibition; rats (permissive hosts) were used as controls in **E, F**

To investigate whether mmu-miR-101b-3p is involved in the progression of meningoencephalitis caused by *A. cantonensis* infection, infected mice underwent AAV-eGFP-mmu-miR-101b-3p tough decoy (TuD) administration (Fig. 2A). After miR-101b-3p inhibition, the brain miR-101b-3p concentration was significantly decreased (Fig. 2B) and body weight loss was significantly alleviated (Fig. 2C). Classical neurological scoring was performed to evaluate the neurologic impairment of mice, and the results revealed neurological severity scores were significantly decreased after miR-101b-3p inhibition (Fig. 2D), while walking abilities were improved after miR-101b-3p inhibition (Supplemental Video S1). In addition, compared to in rats, *A. cantonensis* in the mouse brain was surrounded by inflammatory infiltration, and the inflammatory infiltration around the worms was alleviated after miR-101b-3p inhibition (Fig. 2E). Correspondingly, inflammatory infiltration disrupted the cuticle of *A. cantonensis*, while miR-101b-3p inhibition alleviated the cuticle damage of *A. cantonensis* (similar to as seen with worms derived from rat brains) (Fig. 2F).

### MiR-101b-3p inhibition repressed pyroptosis in the A. cantonensis–infected mouse brain

**Fig. 3:**
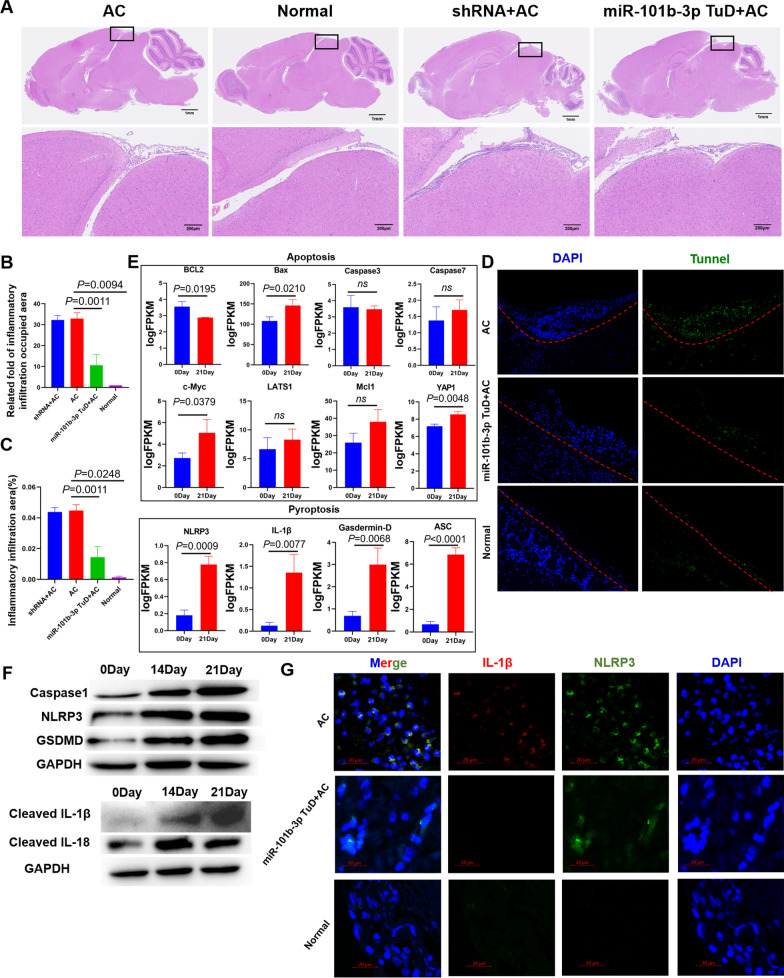
Pyroptosis in the brains of *A. cantonensis*–infected mice was decreased after miR-101b-3p inhibition. **A–C** The inflammatory infiltration caused by *A. cantonensis* infection was decreased after miR-101b-3p inhibition. **D** TUNEL staining indicating cell death in the brain was decreased after miR-101b-3p inhibition. **E** Transcriptome data showed the changes of apoptosis-related genes (*BCL2*, *Bax*, *Caspase3*, *Caspase7*, *c-Myc*, *LATS1*, *Mcl1*, and *YAP1*) and pyroptosis-related genes (*NLRP3*, *IL-1β*, *Gasdermin-D,* and *ASC*). **F** Western blotting revealed that pyroptosis proteins (Caspase1, NLRP3, GSDMD, IL-1β, and IL-18) were up-regulated in the brain after *A. cantonensis* infection. **G** Immunofluorescence showed that NLRP3 and cleaved IL-1β were down-regulated after miR-101b-3p TuD

The inflammation in meningoencephalitis caused by *A. cantonensis* has been well described by researchers. Hematoxylin eosin staining revealed that inflammatory cells and blood cells had infiltrated the pia mater following *A. cantonensis* infection, and miR-101b-3p TuD treatment reduced the cell infiltration area beneath the pia mater (Fig. 3A–C). Studies have shown that inflammation is closely related to cell death [16, 17]. Here, TUNEL staining shown that cell death increased beneath the pia mater (Fig. 3D). TUNEL staining can be used to indicate apoptosis and pyroptosis. RNA sequencing (RNA-seq) data show that apoptosis-related genes (*BCL2*, *Bax*, *Caspase3*, *Caspase7*, *c-Myc*, *LATS1*, *Mcl1*, and *YAP1*) change slightly, while pyroptosis-related genes (*NLRP3*, *IL-1β*, *Gasdermin-D*, and *ASC*) are significantly up-regulated after *A. cantonensis* infection (Fig. 3E). In addition, the up-regulation of pyroptosis-related genes (*Caspase1*, *NLRP3*, *GSDMD*, *IL-1β*, and *IL-18*) in *A. cantonensis*–infected mouse brains were validated by western blotting (Fig. 3F). Interestingly, immunofluorescence showed that NLRP3 and cleaved IL-1β were down-regulated after miR-101b-3p TuD, indicating that the pyroptosis and inflammation caused by *A. cantonensis* infection were alleviated by miR-101b-3p inhibition (Fig. 3G).

### CEBPΑ directly activates pre–miR-101b transcription

**Fig. 4:**
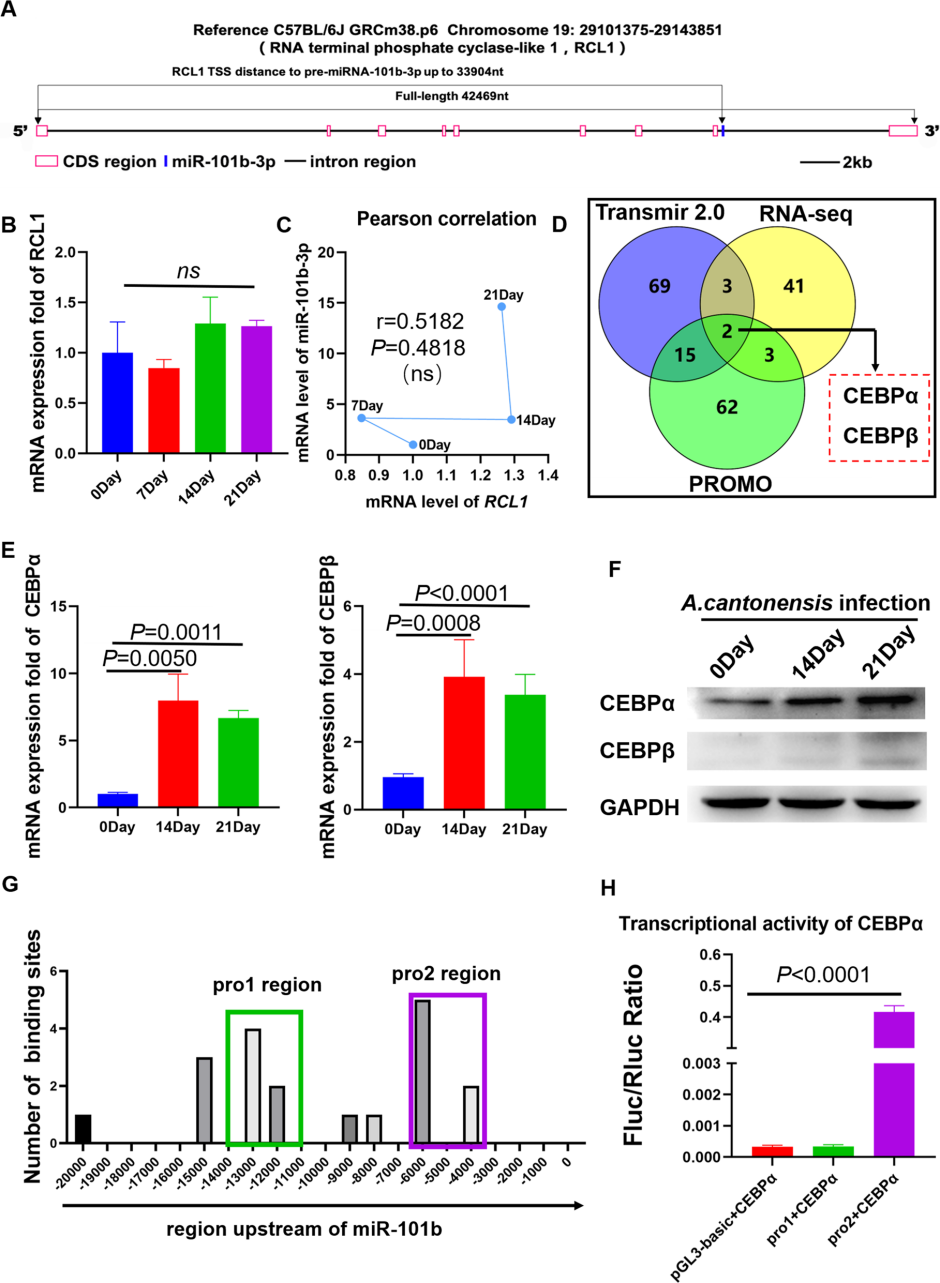
Potential TF screening of miR-101b-3p. **A** miR-101b-3p was located inside the intron of the *RCL1* gene, and the distance from it to the *RCL1* gene TSS was 33,904 nt. **B** *RCL1* was up-regulated slightly after *A. cantonensis* infection. **C** Pearson’s correlation analysis showed that the expressions of miR-101b-3p and *RCL1* are positively associated but not significantly so. **D** Venn diagram for potential TF screening. **E, F** mRNA and protein levels of CEBPα and CEBPβ in the brain after *A. cantonensis* infection. **G** Screening for potential TFBS regions. **H** Dual luciferase assay for transcriptional activity examination of CEBPα

We analyzed the sequence structure of miR-101b. Pre–miR-101b is located on chromosome 19, and Fig. 4A shows that pre–miR-101b is located in an intron of the *RCL1* coding gene. The *RCL1* coding gene consists of 9 exons and 8 introns; the full-length of the coding area is 42,469 nt, while the distance between the *RCL1* transcription start site (TSS) and pre–miR-101b is 33,904 nt. A published report revealed that intron-miRNAs have 2 different patterns of transcriptional activation; either they share a TSS with the host-gene when there is a short TSS distance to the miRNA or they develop an independent locus when the host TSS is far from the miRNA [18, 19]. The up-regulation trend of *RCL1* (Fig. 4B) and that of miR-101b-3p (Fig. 1B) showed a low correlation, indicating that miR-101b may not share a TSS with *RCL1* (Fig. 4C). Since most miRNA TSSs are located within 20,000 bp in front of pre-miRNA, the position of the transcription factor (TF) binding site (TFBS) is highly associated with the TSS; thus, 20,000 bp (GRCm38.p6, chromosome 19: 29115279–29135279) in front of pre–miR-101b was set as a potential location of TFBS, and a 20,000-bp sequence was input into PROMO (http://alggen.lsi.upc.es/cgi-bin/promo_v3/promo/promoinit.cgi?dirDB=TF_8.3#opennewwindow). PROMO was used for TF prediction based on the TFBS motif and finally output 82 potential factors (hereinafter referred to as the PROMO data) (Additional file 1: Fig. S1a). RNA-seq was performed and RNA-seq data were used to analyze different expressions of genes between normal mouse brain tissue and *A. cantonensis*–infected mouse brain tissue; in total, 49 genes of 107 differentially expressed TFs were up-regulated (hereinafter referred to as the RNA-seq data) (Additional file 1: Fig. S1b). The TransmiR database was used to predict potential TF based on open-access CHIP-seq data (TransmiR v2.0 database, http://www.cuilab.cn/transmir), and 104 proteins were selected as potential TFs of mmu-miR-101b (hereinafter referred to as the TransmiR data) (Additional file 1: Fig. S1c). The PROMO data, TransmiR data, and RNA-seq data were used for further screening of potential TFs of miR-101b; as a Venn diagram (Fig. 4D) shows, the area of intersection of the RNA-seq data with the other 2 groups was recognized as containing highly potential TFs due to this portion of genes being actually expressed in the disease model. Among them, CEBPα and CEBPβ had higher mRNA expression levels. In addition, we found that the protein expression level of CEBPα is significantly up-regulated 14 days post-infection and 21 days post-infection, while the CEBPβ expression level did not significantly change (Fig. 4E and F).

**Fig. 5:**
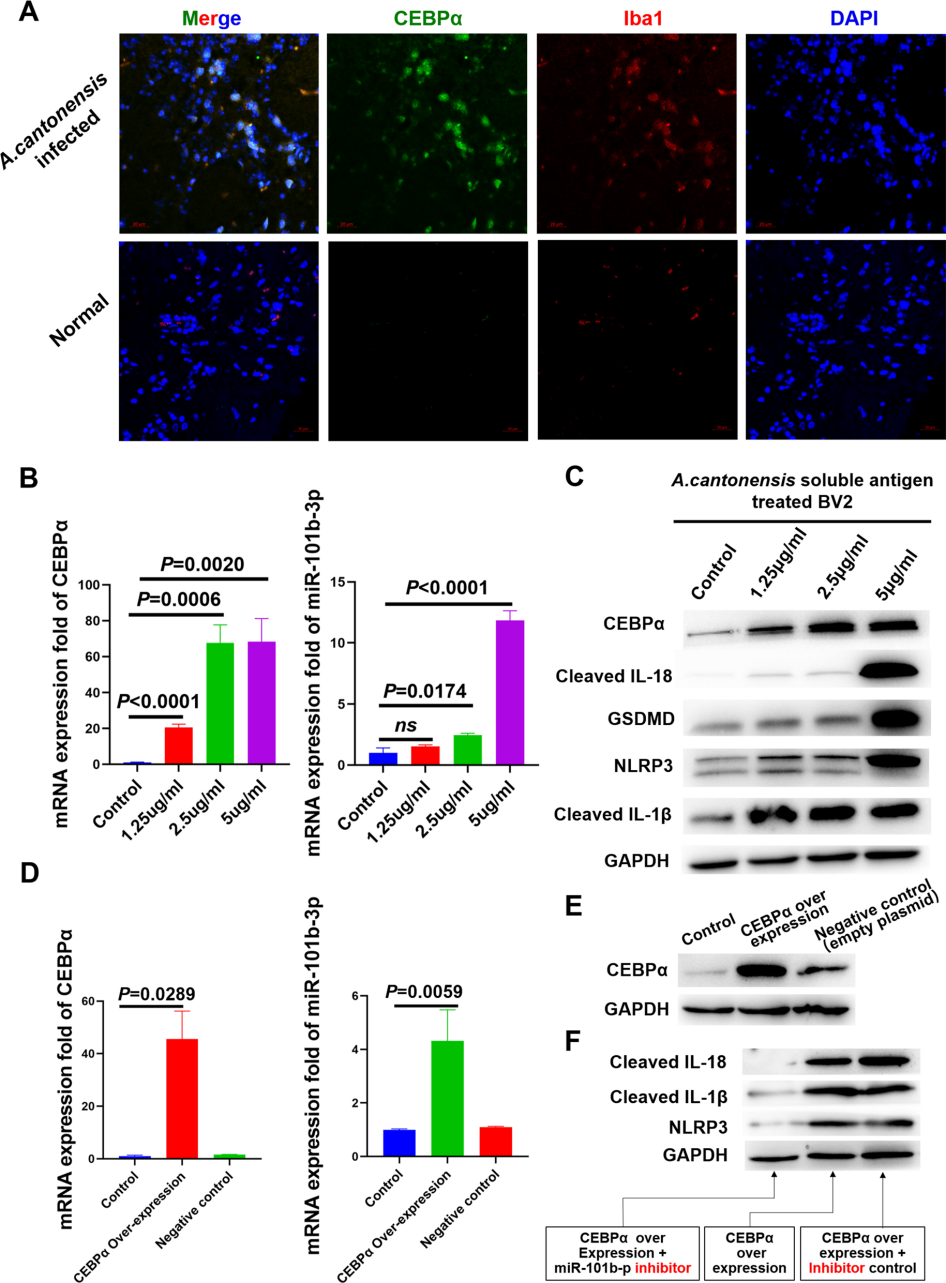
CEBPα promotes pyroptosis in microglia by activating miR-101b. **A** Iba1 was co-localized with CEBPα. **B** Results of CEBPα and miR-101b-3p qPCR after BV2 cell stimulation by *A. cantonensis*–soluble antigen. **C** Expression of CEBPα and pyroptosis-related proteins after BV2 cells were stimulated by *A. cantonensis*–soluble antigen. **D-E** CEBPα over-expression (by carrier plasmids) promoted miR-101b-3p expression. Control group was treated by empty plasmids. **F** CEBPα over-expression lead to expression of pyroptosis, and inhibition of miR-101b-3p repressed CEBPα-induced pyroptosis

To further confirm that CEBPα is the TF of mmu-miR-101b, the 20,000-bp sequence (GRCm38.p6, chromosome 19: 29115279–29135279) in front of pre–miR-101b was input into the JASPAR database (http://jaspar.genereg.net/). As results, based on the sequence logo type of TFBS CEBPα, there were 171 potential TFBSs output by JASPAR, including 19 with scores of ≥ 8 points, which are highly likely to be TFBSs of CEBPα (Fig. 4G). Interestingly, the high-scoring TFBSs predicted by JASPAR were enriched in the − 14-k to − 11-k and − 6-k to − 3.5-k regions upstream of miR-101b; thus 2 sequence fragments, 3,048 bp (chromosome 19: 29121047–29124095) and 2529 bp (chromosome 19: 29129170–29131699), which covered most high-scoring TFBSs, were constructed into Firefly plasmid vectors 1 and 2 (Additional file 1: Fig. S1e) independently, and double fluorescein reporter assay revealed that CEBPα had high transcriptional activity on vector 2 (Fig. 5H). In summary, our results show that CEBPα directly bound to the − 6-k to − 3.5-k region.

### CEBPα promotes microglia pyroptosis by activating miR-101b-3p expression

In an attempt to reveal the cell type expressing CEBPα, immunofluorescence imaging revealed that–Iba1 and CEBPα are co-localized in the mouse brain after *A. cantonensis* infection (Fig. 5A), suggesting that they may expressed by microglia. Then, soluble antigen (1.25, 2.5, and 5 μg/mL) of stage IV *A. cantonensis* was co-cultured with BV2 cells, and qPCR results showed that both CEBPα and miR-101b-3p expression increased with the concentration gradient (Fig. 5B), in accordance with the western blot results of CEBPα (Fig. 5C). In order to confirm whether pyroptosis is mediated by microglia, western blot results showed that pyroptosis-related genes were up-regulated in BV2 cells after *A. cantonensis–*soluble antigen stimulation (Fig. 5C). We further over-expressed CEBPα in the BV2 microglia cell line (Fig. 5D), and the expression of mmu-miR-101b-3p was up-regulated relative to that of the control group (Fig. 5E). To prove that CEBPα promotes microglia pyroptosis by activating miR-101b-3p expression, miR-101b-3p inhibitor and CEBPα were co-transfected into BV2 cells, and western blotting indicated that NLRP3 and cleaved IL-1β and IL-18 were down-regulated in the antagomir treatment group (Fig. 5F). These results suggested that miR-101b-3p inhibition represses pyroptosis in microglia, in accordance with the findings of our mouse in vivo experiment.

## Discussion

**Fig. 6:**
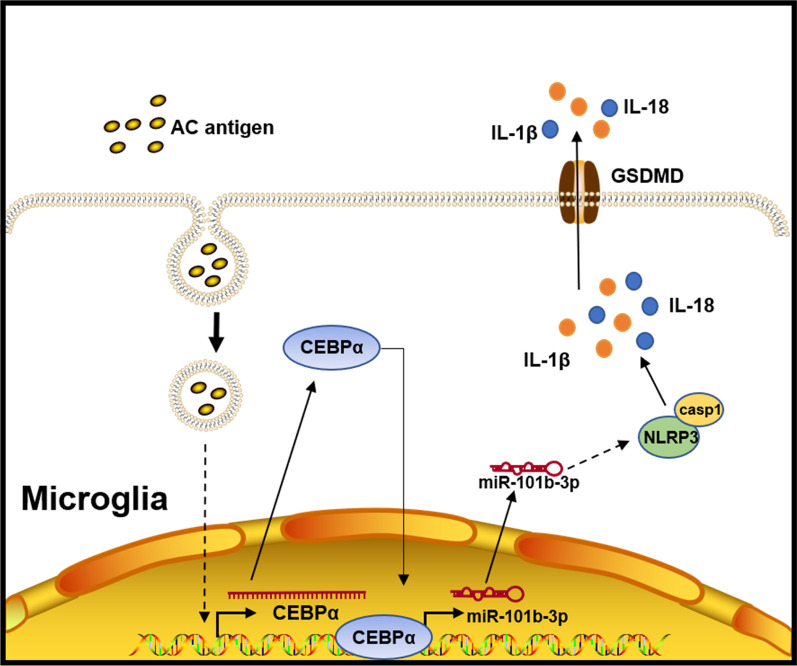
CEBPα promotes pyroptosis in microglia by activating miR-101b-3p expression

*A. cantonensis* dies and degrades in the brains of infected mice (non-permissive hosts); then, *A. cantonensis–*derived antigens trigger an intensive immune response known as meningoencephalitis. Herein, we found that miR-101b-3p is highly expressed in mice infected with *A. cantonensis*. An in vivo inhibition experiment of mice illustrated that miR-101b-3p is a pro-inflammatory factor in meningoencephalitis caused by *A. cantonensis*. After miR-101b-3p inhibition, neurological severity scores were decreased and walking ability was improved, while inflammatory infiltration and blood vessel enlargement were alleviated. Besides, cell death in the mouse brain was decreased, and concentrations of NLRP3 and cleaved IL-1β indicating the presence of pyroptosis and inflammation were reduced. Further, CEBPα was proved to be a TF of miR-101b, and CEBPα was up-regulated in microglia after stimulation by *A. cantonensis*–soluble antigen. Pyroptosis was increased in microglia treated with *A. cantonensis*–soluble antigen, and miR-101b-3p inhibition in BV2 cells repressed pyroptosis mediated by CEBPα. In conclusion, CEBPα promotes pyroptosis in meningoencephalitis induced by *A. cantonensis* infection by activating miR-101b-3p expression (Fig. 6).

miR-101 was reported to be a pro-inflammatory factor [13]; however, the role of miR-101 in *A. cantonensis* infection is unclear. Here, we found that miR-101b-3p was a pro-inflammatory factor in EOMA, and the weight loss and neurological severity scores of *A. cantonensis*–infected mice were significantly alleviated. In addition, miR-101b-3p inhibition alleviated the cuticle damage of *A. cantonensis*. Studies have revealed that miR-101 participates in apoptosis [20] and autophagy [21], but the relationship between miR-101 and pyroptosis is still unclear. Recently, Zhao et al. reviewed the double-edged sword effect of inflammation in cancer progression, i.e., the cancer-inhibiting acute inflammation and cancer-promoting chronic inflammation, and found that inflammasomes mediate pyroptosis, release IL-1β and IL-18, and activate a strong inflammatory response [22]. miR-101 was reported to be expressed in microglia [23] and macrophages [24]. Studies have shown that pro-inflammatory M1 immunity exacerbates tissue damage [25] while anti-inflammatory/regulatory M2 immunity promotes inflammation resolution and tissue repair [26], and microglia/macrophages exert an important role in *A. cantonensis* infection [27, 28]. Other studies have shown that inflammation is closely related to cell death, especially pyroptosis [16, 17, 29, 30]. Inflammasomes are necessary for pyroptosis; as an inflammasome sensor, the Nod-like receptor protein NLRP3 can be activated by viral nucleic acid, microbial toxins, and bacterial surface components [31]. He et al. [32] reported that NLRP3-induced microglial pyroptosis was important for HIV-1 envelope protein gp-120 induced neuroinflammation and neuropathology. Microglia NLRP3 inflammasome also contributed to APP/PS1 mice Aβ accumulation, which was associated with improved neuronal function [33]. In this research, we found that cell death beneath the pia mater was increased after *A. cantonensis* infection, and pyroptosis-related genes in the mouse brain were significantly up-regulated after *A. cantonensis* infection. Interestingly, NLRP3 and cleaved IL-1β were down-regulated after miR-101b-3p TuD, indicating that pyroptosis and inflammation caused by *A. cantonensis* infection were alleviated by miR-101b-3p inhibition.

Studies have shown that there is a window for albendazole and mebendazole efficacy in the treatment of *A. cantonensis* [2]. The early use of these drugs to achieve a parasite-elimination effect is significant, and albendazole can reduce brain inflammation in *A. cantonensis* infection, but some studies have found that these drugs may aggravate central nervous system symptoms [5]. Glucocorticoid immunosuppressants are also commonly used in the treatment of *A. cantonensis* [34], but long-term high-dose use will bring serious side effects [35]. In this paper, we testified that miR-101b-3p blocking received alleviation of the inflammation in mouse brains and neurological symptoms; in order to develop a potential target to regulate miR-101b-3p, the TF of miR-101b is the most promising drug target candidate. The AP-1/miR-101-2 feedback loop was illustrated in hepatoma cells to prevent cancer metastasis [36]. However, the upstream regulation mechanism of miR-101 expression in *A. cantonensis* infection has not been elucidated.

CEBPα inhibition by siRNA intensely suppressed lipopolysaccharide-induced inflammatory factors, suggesting that CEBPα promotes inflammatory factor secretion of disease-associated microglia [37]. The tumor-suppressive effects of CEBPα—including the prevention of epithelial-to-mesenchymal transition [38], regulation of lineage-specific gene expression, induction of growth arrest [39], and inhibition of tumor proliferation [40]—have garnered wide concern as these anti-tumor effects may related to the pro-inflammatory effect of CEBPα. During experimental autoimmune encephalomyelitis, miR-124 deactivated macrophages by targeting CEBPα [41]. Another study illustrated that miR-124 promotes microglia M2 polarization by targeting CEBPα [42], indicating that CEBPα is a key regulator of macrophages/microglia. Besides, CEBPα plays an important role in miRNA expression promotion or inhibition. CEBPα blocks miR-182 by directly binding to the promoter region, leading to impaired human granulocytic differentiation [43]. CEBPα cooperating with Sp1 was reported to induce miR-122 expression by binding to its promoter in hepatocellular carcinoma cells [44], and miR-122 is a potential therapeutic target for the treatment of liver disease [45]. CEBPα promotes miR-223 expression to exert an effect in human granulopoiesis [46], and the CEBPα/miR-223 axis in neutrophils was further proven to reduce the susceptibility to alcohol-induced liver injury [47]. We found that CEBPα can activate miR-101b-3p expression after *A. cantonensis* infection; according to miRNA target prediction, CEBPα is a potential target of miR-101b-3p, and CEBPα/miR-101b-3p may form a negative feedback loop. We also found that CEBPα can be expressed by microglia, and the expressions of CEBPα and miR-101b-3p in BV2 cells were increased after treatment with the soluble antigen of stage IV *A. cantonensis*. NLRP3 and cleaved IL-1β and IL-18 in BV2 cells were down-regulated after miR-101b-3p inhibition and CEBPα was co-transfected. Our results suggest that CEBPα promotes microglia pyroptosis by activating miR-101b-3p expression.

## Conclusion

In conclusion, we found that miR-101b-3p inhibition alleviates inflammation infiltration and pyroptosis in *A. cantonensis* infection. In addition, we found that CEBPα directly binds to the − 6-k to − 3.5-k region upstream of miR-101b and activates miR-101b-3p expression in microglia. These data suggest a novel CEBPα/miR-101b-3p/pyroptosis pathway in *A. cantonensis* infection. Our study also indicates that the CEBPα/miR-101b-3p/pyroptosis pathway contributes to the inflammation inducted by *A. cantonensis* infection, and both CEBPα and miR-101b-3p may be potential therapeutic targets for treating *A. cantonensis* infection.

## Supplementary Information

**Additional file 1**. Potential transcription factor prediction.

## Data Availability

All data generated or analysed during this study are included in this published article.

## Abbreviations

CEBPα: CCAAT/enhancer-binding protein alpha
RT-PCR: Real-time polymerase chain reaction
DAPI: 4′,6-Diamidino-2-phenylindole
TuD: Tough decoy
TSS: Transcription start site
TFBS: Transcription factor binding site
