# Supplementary material for: CEBPα/miR-101b-3p promotes meningoencephalitis in mice infected with Angiostrongylus cantonensis by promoting microglial pyroptosis
Source: Cell Commun Signal. 2023 Feb 6;21:31. doi: 10.1186/s12964-023-01038-y (PMC9903543; doi:10.1186/s12964-023-01038-y)

Supplementary data

Figure.s1a PROMO prediction base on sequence


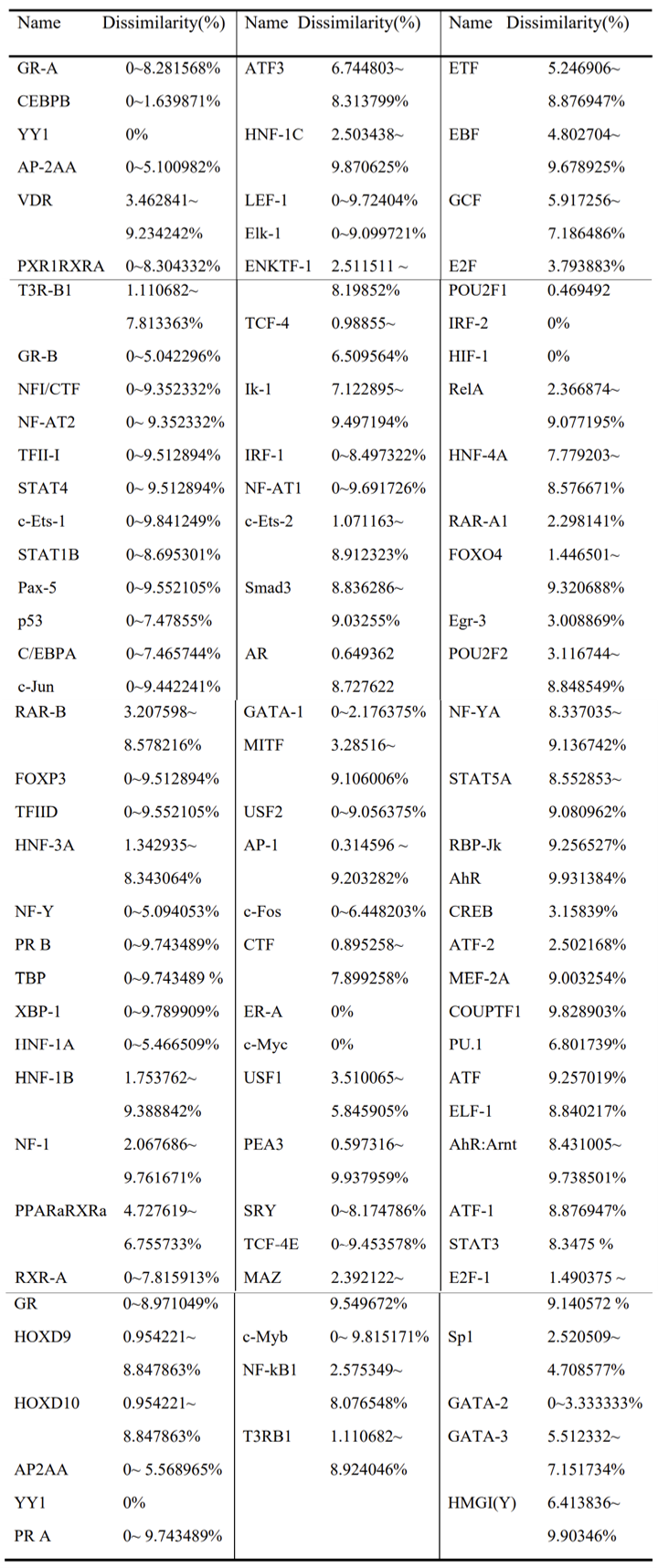


Figure.s1b RNA-seq data of up-regulated transcription factor


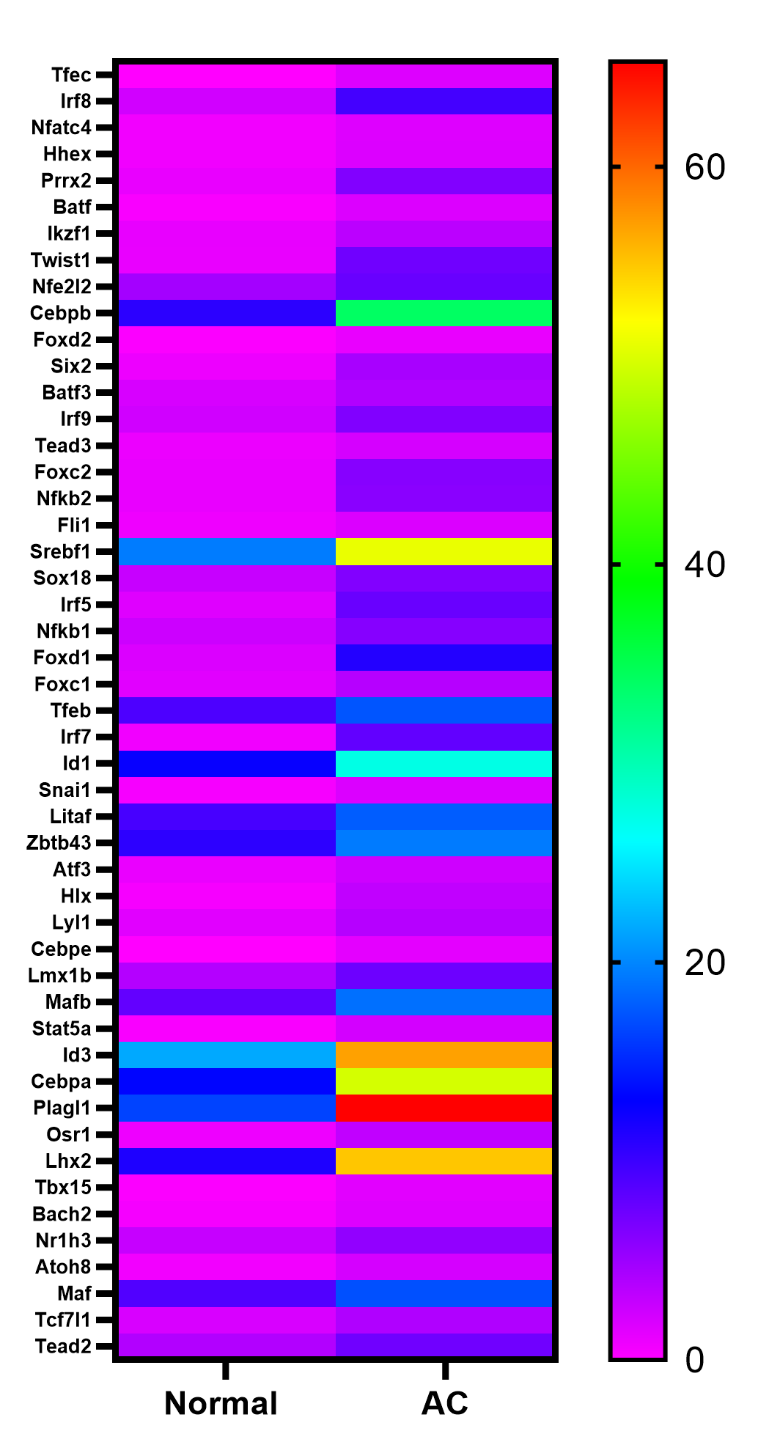


Figure.s1c Transcription factor predicted by TransmiR in *A.cantonensis* infection


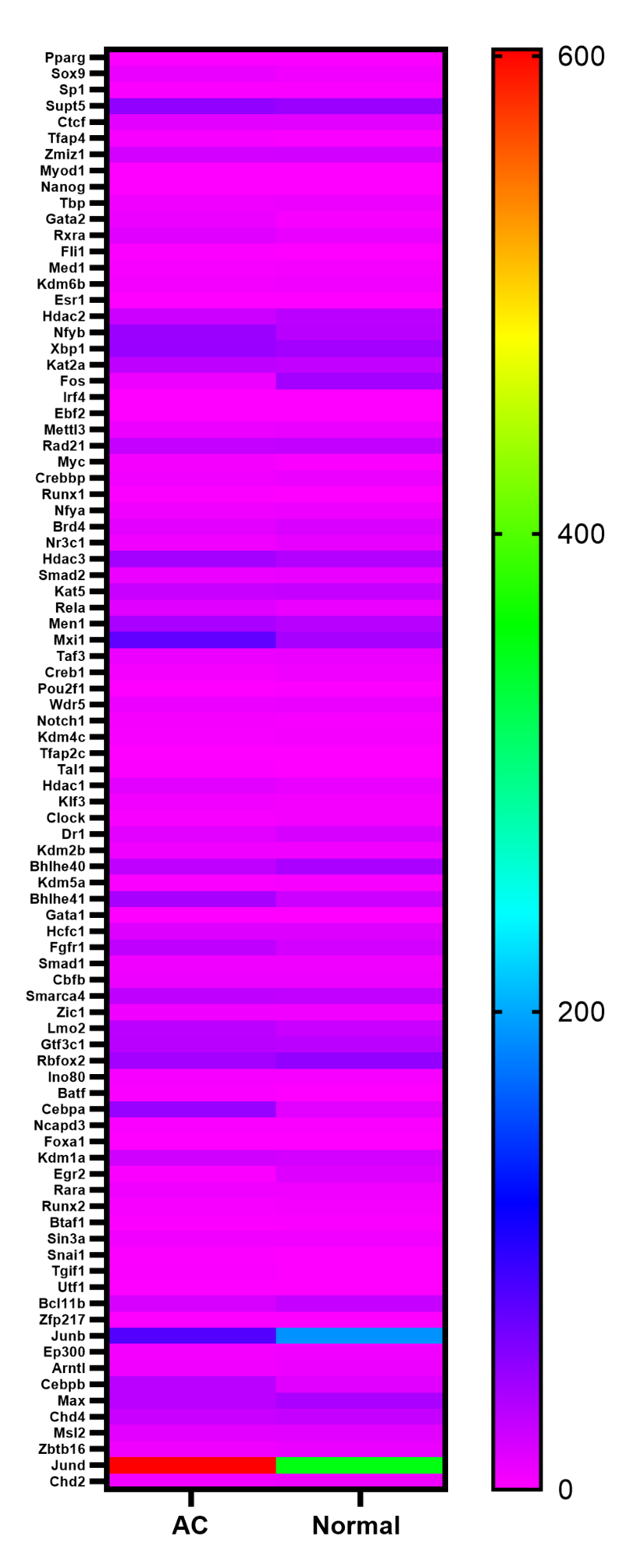

Supplement: Supplementary file 2 — Additional file 1. Potential transcription factor prediction. [file 12964_2023_1038_MOESM2_ESM.docx]
